# Molecular imaging of inflammation crosstalk along the cardio-renal axis following acute myocardial infarction

**DOI:** 10.7150/thno.61423

**Published:** 2021-07-06

**Authors:** Rudolf A. Werner, Annika Hess, Tobias Koenig, Johanna Diekmann, Thorsten Derlin, Anette Melk, James T. Thackeray, Johann Bauersachs, Frank M. Bengel

**Affiliations:** 1 Department of Nuclear Medicine, Hannover Medical School, Hannover, Germany.; 2 Department of Cardiology and Angiology, Hannover Medical School, Hannover, Germany.; 3 Department of Kidney, Liver and Metabolic Diseases, Children's Hospital, Hannover Medical School, Hannover, Germany.

**Keywords:** Cardiorenal syndrome, myocardial infarction, molecular imaging, CXCR4, inflammation

## Abstract

**Rationale:** Acute myocardial infarction (MI) triggers a systemic inflammatory response including crosstalk along the heart-kidney axis. We employed radionuclide-based inflammation-targeted whole-body molecular imaging to identify potential cardio-renal crosstalk after MI in a translational setup.

**Methods:** Serial whole-body positron emission tomography (PET) with the specific CXCR4 ligand ^68^Ga-Pentixafor was performed after MI in mice. Tracer retention in kidneys and heart was compared to hematopoietic organs to evaluate systemic inflammation, validated by *ex vivo* analysis and correlated with progressive contractile dysfunction. Additionally, 96 patients underwent ^68^Ga-Pentixafor PET within the first week after MI, for systems-based image analysis and to determine prognostic value for adverse renal outcome.

**Results:** In mice, transient myocardial CXCR4 upregulation occurred early after MI. Cardiac and renal PET signal directly correlated over the time course (r = 0.62, p < 0.0001), suggesting an inflammatory link between organs. *Ex-vivo* autoradiography (r = 0.9, p < 0.01) and CD68 immunostaining indicated signal localization to inflammatory cell content. Renal signal at 7d was inversely proportional to left ventricular ejection fraction at 6 weeks after MI (r = -0.79, p < 0.01). In patients, renal CXCR4 signal also correlated with signal from infarct (r = 0.25, p < 0.05) and remote myocardium (r = 0.39, p < 0.0001). Glomerular filtration rate (GFR) was available in 48/96 (50%) during follow-up. Worsening of renal function (GFR loss >5 mL/min/1.73m^2^), occurred a mean 80.5 days after MI in 16/48 (33.3%). Kaplan-Meier analysis revealed adverse renal outcome for patients with elevated remote myocardial CXCR4 signal (p < 0.05). Multivariate Cox analysis confirmed an independent predictive value (relative to baseline GFR, LVEF, infarct size; HR, 5.27).

**Conclusion:** Systems-based CXCR4-targeted molecular imaging identifies inflammatory crosstalk along the cardio-renal axis early after MI.

## Introduction

It is increasingly recognized that organ systems do not operate independently, such that injury to one organ can bear grave consequences for disparate organs 1. One example of this concept is the cardiorenal syndrome, comprising a myriad of disorders, and exemplifying the bidirectional nature of pathophysiologic interaction between failing heart and kidney 2. Renal deterioration is an independent risk factor of all-cause mortality among heart failure patients 3. Likewise, patients with primary renal disease die more frequently of cardiovascular cause than due to kidney failure itself 4. Moreover, functional improvement in one organ is also linked to better performance of the other, as exemplified e.g. by the reversal of cardiac dysfunction after kidney transplantation in patients afflicted with uremic cardiomyopathy 5. While biologic mechanisms underlying this interaction remain unclear, several afterload-independent pathways have been identified in cardiorenal syndrome 2. One implicated pathway is chronic inflammation which thought to play a prominent role in initiating and exacerbating both cardiac and renal impairment 2, 5.

A persistent inflammatory response after acute myocardial infarction (AMI) contributes to adverse left ventricular (LV) remodeling, independent of infarct size 6. Similarly, circulating inflammatory cytokines in chronic heart failure are linked with adverse outcome 7 and drugs neutralizing the inflammatory pathways effectively lower cardiovascular events among patients after myocardial infarction 8, 9. After primary damage to the myocardium, the kidneys are also prone to leukocyte infiltration 10, which may contribute to renal injury in the framework of cardiorenal syndrome 11. Immune-system mediated crosstalk along the cardiorenal axis, its temporal dynamics and its relevance for subsequent organ dysfunction have not yet been fully elucidated 2.

We hypothesized that AMI would evoke parallel inflammation of the damaged region and kidneys. Using a molecular positron emission tomography (PET) imaging platform, we serially investigated total body inflammation to gain insights into heart-kidney crosstalk after primary cardiac injury in mice after coronary artery ligation and patients after first ST elevation myocardial infarction.

## Methods

### Animal model and experimental protocols

The local state authority approved all animal procedures (Niedersächsisches Landesamt für Verbraucherschutz und Lebensmittelsicherheit) and animal protocols were conducted in accordance to guiding principles of the American Physiological Society. C57BL/6N mice (Charles River, 24 ± 2 g) underwent permanent left anterior descending coronary artery ligation, as previously described 12. Parts of this retrospective cohort have been described previously and data were newly analyzed for the purpose of this project 13.

### Whole-body small animal PET

Serial PET imaging was conducted using a dedicated small animal PET scanner (Inveon DPET, Siemens, Knoxville, Tennessee) at 1 day, 3 days, 7 days and 6 weeks after surgery, as previously described 14. The majority of animals (n=19) have been imaged at every single of the first three time-points. Animals that died of LV rupture at 5-7 days post-MI were substituted by additional mice at MI+7d. The total number of animals for every single time-point was as follows: MI+1d, n = 23; MI+3d, n = 23; MI+7d, n = 24 and MI+6wk, n = 11. In every of those mice, infarct, remote myocardium and spleen could be identified, whereas the kidneys could be analyzed in the following number of animals: MI+1d, n = 19; MI+3d, n = 20; MI+7d, n = 22 and MI+6wk, n = 11. For imaging of leukocyte infiltration, the CXC-motif chemokine-receptor 4 (CXCR4) targeted radiotracer ^68^Ga-Pentixafor (10 ± 1.4 MBq) was administered as a 0.1 to 0.15 ml bolus via the tail vein, under isoflurane anesthesia. A 10 min static scan was acquired at 50 min post-injection. Then, ^18^F-fluorodeoxyglucose (^18^F-FDG) was injected for anatomic co-localization of the heart and infarct territory, and a second static 10 min scan was conducted after 30 min of continued isoflurane. Image reconstruction and volume-of-interest (VOI) analysis were done as previously described 14. Organ localization was aided by anatomic guides with merged computed tomography images 1. Mean percent injected dose per gram (%ID/g) values were calculated for infarct, remote myocardium, spleen and both kidneys, as done previously 15.

### Cardiac magnetic resonance (CMR)

For assessment of LV function at 6 wks after MI, CMR was performed using a dedicated small animal 7T MR system (PharmaScan 70/16, Bruker BioSpin GmbH, Erlangen, Germany), as described previously 12. Images were reconstructed to 10 frames for each cardiac cycle.

### *Ex-vivo* tissue analysis

To confirm tracer distribution and assess inflammatory cell infiltration, *ex vivo* autoradiography and immunohistochemistry were performed in a subset of mice at 1 day, 3 days, 7 days and 6 weeks after surgery (n = 2 each). Briefly, at the conclusion of PET image acquisition, mice were sacrificed and the heart and kidneys were excised. Tissues were snap frozen in OCT compound (Tissue Tec) under liquid nitrogen, and sections in the short axis of the heart or coronal axis of the kidneys were taken using a cryostat (Leica) and adjacent sections were allocated for autoradiography (10 µm) and histopathology (6 µm). For autoradiography, sections were immediately exposed to a phosphor screen (PerkinElmer multisensitive) for 60min. The exposed images were then digitalized for analysis (Cyclon). Immunostaining for macrophages was performed as described previously 16. Briefly, after fixation in acetone (10min), sections were sequentially blocked for endogenous biotin and avidin (Dako blocking kit) and non-specific binding in 10% horse serum. Sections were then incubated for 60min with biotin-conjugated rat anti-mouse CD68 (BioRad MCA1957B, clone FA-11), followed by streptavidin peroxidase, and visualization with diaminobenzidine. Sections were counterstained with Mayer's hematoxylin. Overview light microscopy images were acquired using a stereomicroscope at 2.5x magnification (Leica DM LB2, Leica Microsystems GmbH, Nussloch, Germany).

### Patients

Between January 2015 and February 2018, 96 consecutive patients (14 females; 62 ± 11 yrs) have been enrolled in a retrospective setting. All subjects had undergone resting myocardial perfusion single-photon emission computed tomography (SPECT) and inflammation-targeted ^68^Ga-Pentixafor PET within median 4d after reperfusion therapy for AMI at Hannover Medical School (MHH). All patients had been treated by percutaneous coronary intervention and stenting within a median of 3h of symptom onset (range, 1 h - 4 d). Eligibility criteria included the diagnosis of ST-segment elevation myocardial infarction (STEMI), absence of a prior history of AMI or coronary intervention or other cardiac procedures, success of reperfusion immediately after angioplasty, absence of systemic immunologic or infectious disease, and availability of complete imaging datasets, clinical records and laboratory parameters at the time of reperfusion therapy. As determined by standard MHH clinical procedures, baseline glomerular filtration rate (GFR, mL/min/1.73 m^2^) was assessed. The local ethical committee was informed of data analysis for the purpose of this study and agreed to the project (protocol #2820-2015).

### Non-invasive clinical imaging

Electrocardiographically gated perfusion SPECT was obtained at rest using 361 ± 43 MBq ^99m^Tc-Sestamibi and a dedicated cardiac camera (GE Discovery 530c, GE Healthcare, Waukesha, Wisconsin). Infarct size (summed rest perfusion score) was determined using a 60% threshold of maximal activity from perfusion SPECT polar maps. LV ejection fraction (LVEF) was determined from gated SPECT. ^68^Ga-Pentixafor was synthesized according to good manufacturing practice as previously described 17, using an automated module and CPCR4.2 provided by PentixaPharm (Fürstenfeldbruck, Germany). Static PET images were acquired for 20 min using a Biograph mCT 128 scanner (Siemens, Knoxville, Tennessee), starting at 60 min after injection of ^68^Ga-Pentixafor (121 ± 29 MBq). On ^68^Ga-Pentixafor PET, spherical VOIs were set on infarcted and remote myocardium (guided by SPECT images), the spleen, the blood pool and the upper pole of the renal cortex (carefully avoiding the renal pelvis), to derive peak standardized uptake values (SUV). If both kidneys were in the field-of-view, the mean SUV of both sides was calculated (n = 70/96, 72.9%), whereas in 9/96 cases the right (9.4%) and in 17/96 (17.7%) subjects, only the left kidney was visible for analysis.

### Worsening renal function

In all 96 patients (100%), renal function was assessed at baseline by measuring the GFR 18. After a mean follow-up of 8.6 ± 11.3 mo (range, 22 days - 51 months), GFR was available in 48/96 (50%). We defined worsening of renal function as an annual decline in GFR of >5 mL/min/1.73 m^2^, based o*n Kidney Disease: Improving Global Outcome Society (KDIGO)* 19.

### Statistical analysis

Statistical analyses were performed using R (version 3.6.1, R Core Team, 2019, Vienna, Austria) and Prism (version 8.4.3, GraphPad, San Diego, California). Quantitative variables are expressed as mean ± standard deviation (if normally distributed), or as median and range (if not normally distributed). For comparison of three or more groups, one-way or two-way ANOVA with Bonferroni's post hoc was used. Pearson's correlation was used to determine the association between normally distributed clinical and imaging variables. The 2-tailed paired or unpaired Student's t-test was used to compare normally distributed variables within the entire group, or between subgroups with and without worsening renal function, respectively. Cutoffs for the prediction of worsening renal function-free survival were determined by receiver operating characteristics (ROC) analysis, using the Youden Index for maximization of specificity and sensitivity. Univariate Kaplan-Meier analysis was then performed using thresholds established by ROC, and the nonparametric log-rank test was used to determine significant outcome differences between subgroups. Finally, multivariate Cox regression analysis was applied to determine independent predictors of worsening renal function 20. A *p*-value of *<0.05* was considered to be statistically significant.

## Results

### Myocardial infarction evokes parallel transient increase in cardiac and renal CXCR4 signal

**Figure 1:**
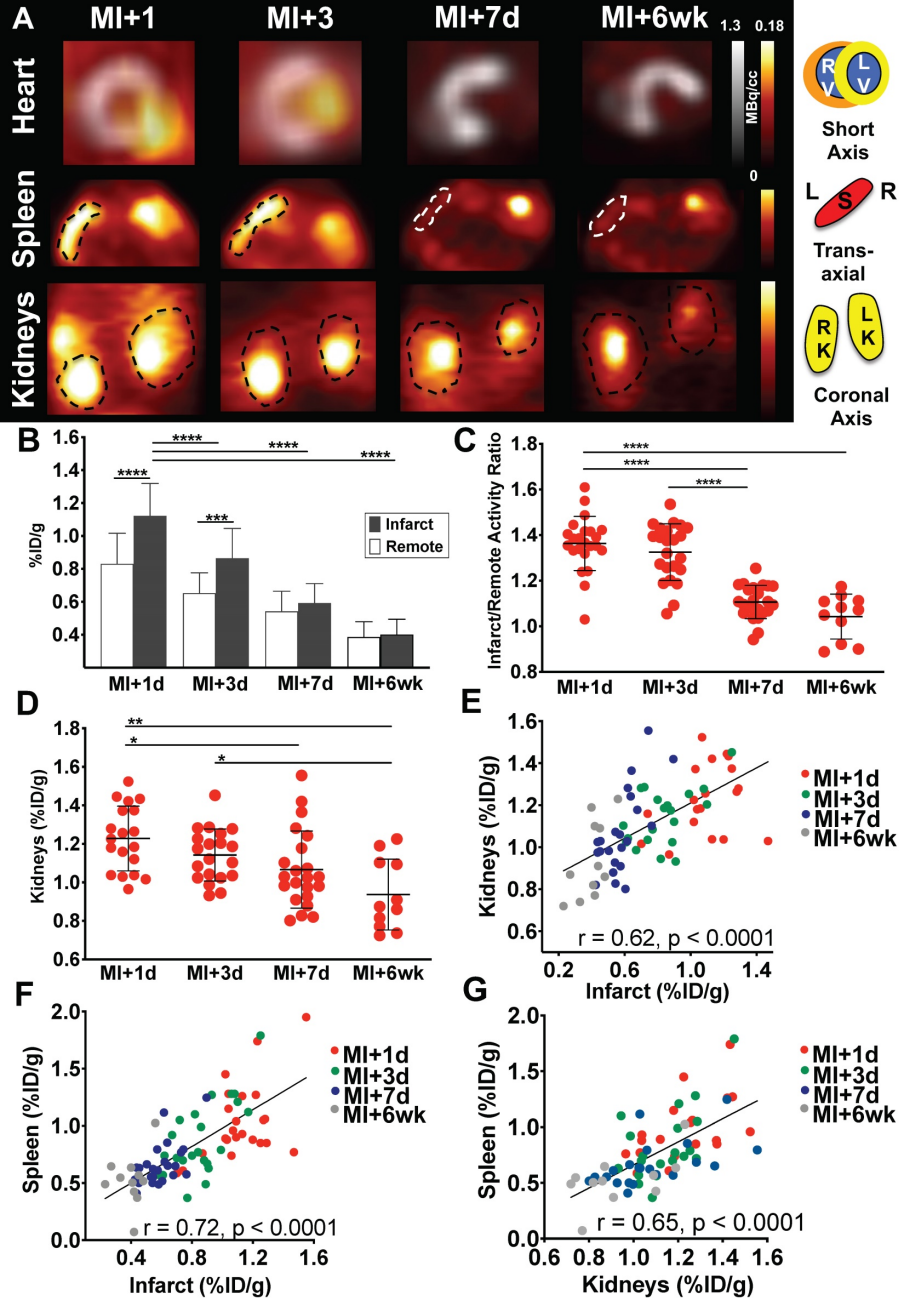
(A) Representative ^68^Ga-Pentixafor PET images, fused with concomitant^ 18^F-fluorodeoxyglucose images of the myocardium after myocardial infarction (MI) in mice (short-axis). ^68^Ga-Pentixafor PET images of the spleen (transaxial) and kidneys (coronal axis) are also displayed. (B) Quantitative assessment of myocardial ^68^Ga-Pentixafor %ID/g, (C) infarct to remote activity ratio and (D) renal ^68^Ga-Pentixafor %ID/g. In all animals, infarct-derived signal was directly correlated with (E) kidney and (F) splenic signal. The latter signal was also significantly associated with renal CXCR4 expression (G). (B-G: MI+1d, n = 23; MI+3d, n = 23; MI+7d, n = 24 and MI+6wk, n = 11). RV, right ventricle; LV, left ventricle; S, spleen; L, Left; R, Right; RK, right kidney; LK, left kidney. * p < 0.05, ** p < 0.001, *** p = 0.0001, **** p < 0.0001.

Consistent with prior observations 12, mice demonstrated a distinctly elevated CXCR4 signal in the infarct region at day 1, followed by a gradual decline from day 3 through day 7 and week 6 (**Figure 1A**). ^68^Ga-Pentixafor uptake in the infarct declined by 23.2% from day 1 to day 3 (p < 0.0001), to 47.3% at day 7 (p < 0.001), to a maximal decline of 64.3% at 6 weeks (p < 0.0001 *vs.* MI+1d, **Figure 1B**). Infarct/remote ratio was highest early after coronary ligation (1.36 ± 0.12 at day 1, 1.33 ± 0.12 at day 3, p = 0.99), and declined significantly at day 7 (1.11 ± 0.07, p < 0.0001) and 6 weeks (1.04±0.1, p < 0.0001 *vs.* MI+1d) (**Figure 1C**). Similar to CXCR4 signal in the infarct territory, renal ^68^Ga-Pentixafor uptake declined by 7.3% from day 1 to day 3 (p = 0.8), to 13% at day 7 (p < 0.05), to a maximal decline of 23.7% at 6 weeks (p < 0.001 *vs.* MI+1d) (**Figure 1D**). Across all time points, CXCR4 signals from infarcted myocardium and kidneys were directly correlated (r = 0.62, p < 0.0001) (**Figure 1E**). To evaluate systemic immune activation in response to myocardial infarction, we measured CXCR4 signal from the spleen as a hematopoietic reservoir. Similar to heart and kidney, the highest CXCR4 PET signal was observed at 1d after infarct, with a gradual decline over 7d, and further decrease at 6wk (p < 0.001 *vs.* MI+1d). The signal in the spleen also correlated directly with the infarct (r = 0.72, p < 0.0001, **Figure 1F**) and kidneys (r = 0.65, p < 0.0001) (**Figure 1G**).

### Elevated CXCR4 signal corresponds to increased macrophage density in infarct territory and kidneys

**Figure 2:**
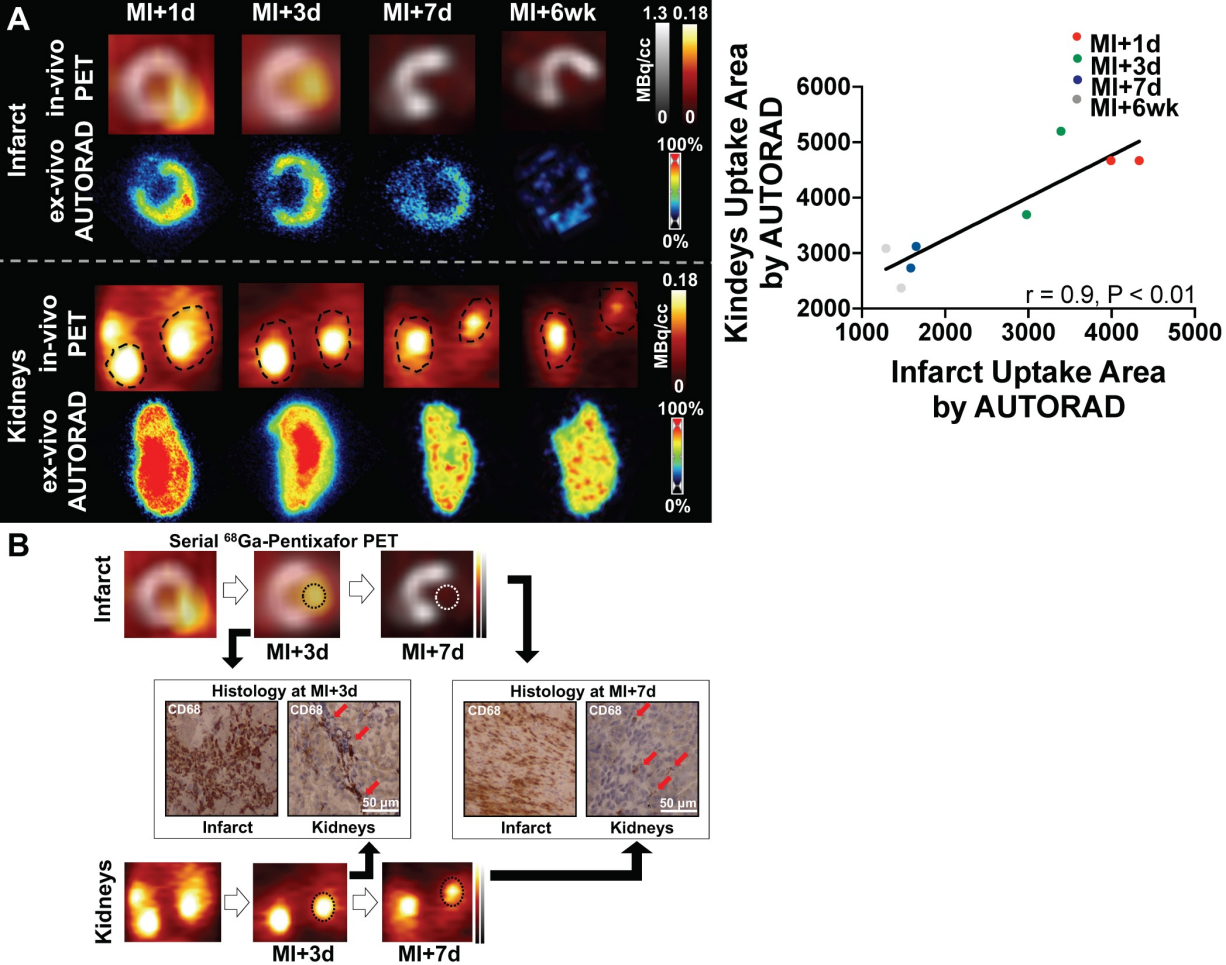
(A)** Left:** *Upper rows:* In mice after myocardial infarction (MI), *in-vivo* signal of CXCR4-directed PET declined over time in the infarct territory (1st row), which was confirmed by *ex-vivo* autoradiography (AUTORAD) of the heart (2nd row, short-axis). *Lower rows:* Findings were paralleled by the in- and *ex-vivo* signal of the kidneys (coronal axis). **Right:** Quantitative analysis demonstrated a strong correlation between *ex-vivo* AUTORAD sections of heart and kidneys. (B) Paralleled by the *in-vivo* CXCR4-directed PET signal, CD68 staining in the infarct territory and kidneys confirmed a decrease in monocyte content from day 3 post-MI (MI+3d) to MI+7d (red arrows; n = 2).

To validate the selectivity of CXCR4 PET signal for inflammation over clearance in the kidney, *ex-vivo* autoradiography was performed in cardiac and renal sections. Radioactivity regional distribution confirmed *in vivo* spatiotemporal pattern in the myocardial infarct territory and kidney parenchyma (**Figure 2A**). *Ex-vivo* analysis validated increased signal in the infarct relative to the remote myocardium, which declined over 7d and 6wk after injury. Moreover, *ex vivo* autoradiography signal intensity in the kidney was similar to the *in-vivo* PET images across all time points, supporting the accuracy of imaging. Quantitative analysis demonstrated a strong correlation between *ex-vivo* autoradiography sections of heart and kidneys (r = 0.9, p < 0.01, **Figure 2A**). CD68 immunostaining in the infarct territory and renal parenchyma identified increased density of macrophages in both organs at MI+3d, which was less prominent at MI+7d (**Figure 2B**), thereby suggesting image-based identification of tissue inflammation in both organs.

### Renal CXCR4 signal is associated with adverse cardiac functional outcome in mice

**Figure 3:**
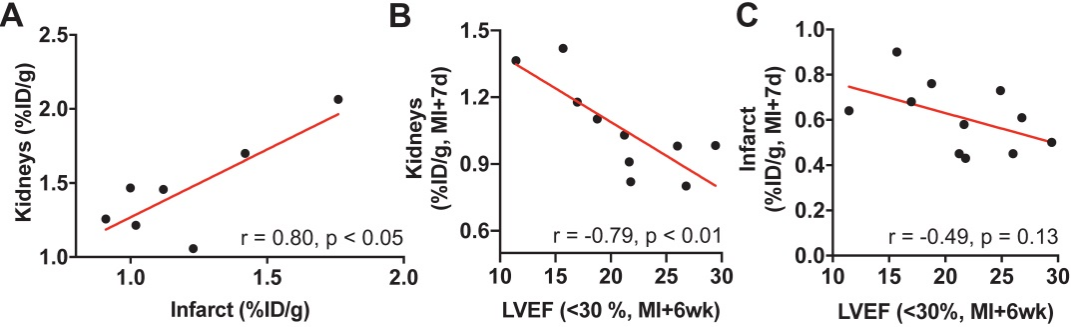
(A) Among mice that died of left ventricular rupture, renal and infarct-derived CXCR4 signal was strongly correlated (r = 0.80, p < 0.05, n=7). (B) In surviving animals with more severe contractile impairment (left ventricular ejection fraction (LVEF) < 30%)), CXCR4 signal intensity in the kidneys at 7 days after the infarct (MI+7d) was inversely proportional to cardiac function at 6 weeks after MI (MI+6wk; n = 10). (C) The association of the infarct-derived CXCR4 signal at MI+7d with later cardiac functional outcome (MI+6wk) was comparatively less pronounced (n = 11).

7 mice died of LV rupture 5-7d after coronary artery ligation as an indicator of impaired infarct healing. Consistent with prior work 12, these animals showed sustained cardiac inflammation at 3 days post-MI compared to survivors (%ID/g: 1.21 ± 0.3 *vs.* 0.87 ± 0.18, p < 0.001). Of note, renal CXCR4 signal was also higher at day 3 among mice with subsequent LV rupture compared to survivors (%ID/g: 1.46 ± 0.34 *vs.* survivors, 1.14 ± 0.13, p < 0.05). Among mice that died of LV rupture, renal and infarct-derived CXCR4 signal was strongly correlated (r = 0.80, p < 0.05; **Figure 3A**).

Surviving animals developed variable contractile dysfunction 6 weeks after infarction. We subdivided these animals into severe (LVEF<30%) and moderate (LVEF>30%) functional impairment at 6wk. In animals with severe LV dysfunction cardiac or renal signal obtained at MI+1d (r = -0.12, r = 0.30; **Figure S1A-B**) or MI+3d (r = -0.52, r = 0.05**; Figure S1C-D**) did not reach significance with late cardiac function at 6 wk (p ≥ 0.16, respectively). CXCR4 signal intensity in the kidneys 7d after infarction, however, was inversely proportional to late cardiac function (r = -0.79, p < 0.01, **Figure 3B**). Conversely, mice with modest cardiac dysfunction displayed no correlation between subacute kidney signal and late ejection fraction (r = -0.1, p = 0.8**; Figure S1E**). Moreover, in animals with severe LV dysfunction, renal CXCR4 signal at MI+7d emerged as a stronger predictor for later adverse cardiac functional outcome relative to the infarct signal at MI+7d (r = -0.49, p = 0.13, **Figure 3C**), confirming relevance of CXCR4 signaling in cardio-renal interaction and potentially reflecting the different timecourse of inflammation in both organs.

### Systemic cardio-renal network analysis by CXCR4-targeted imaging can be translated to patients after AMI

**Figure 4:**
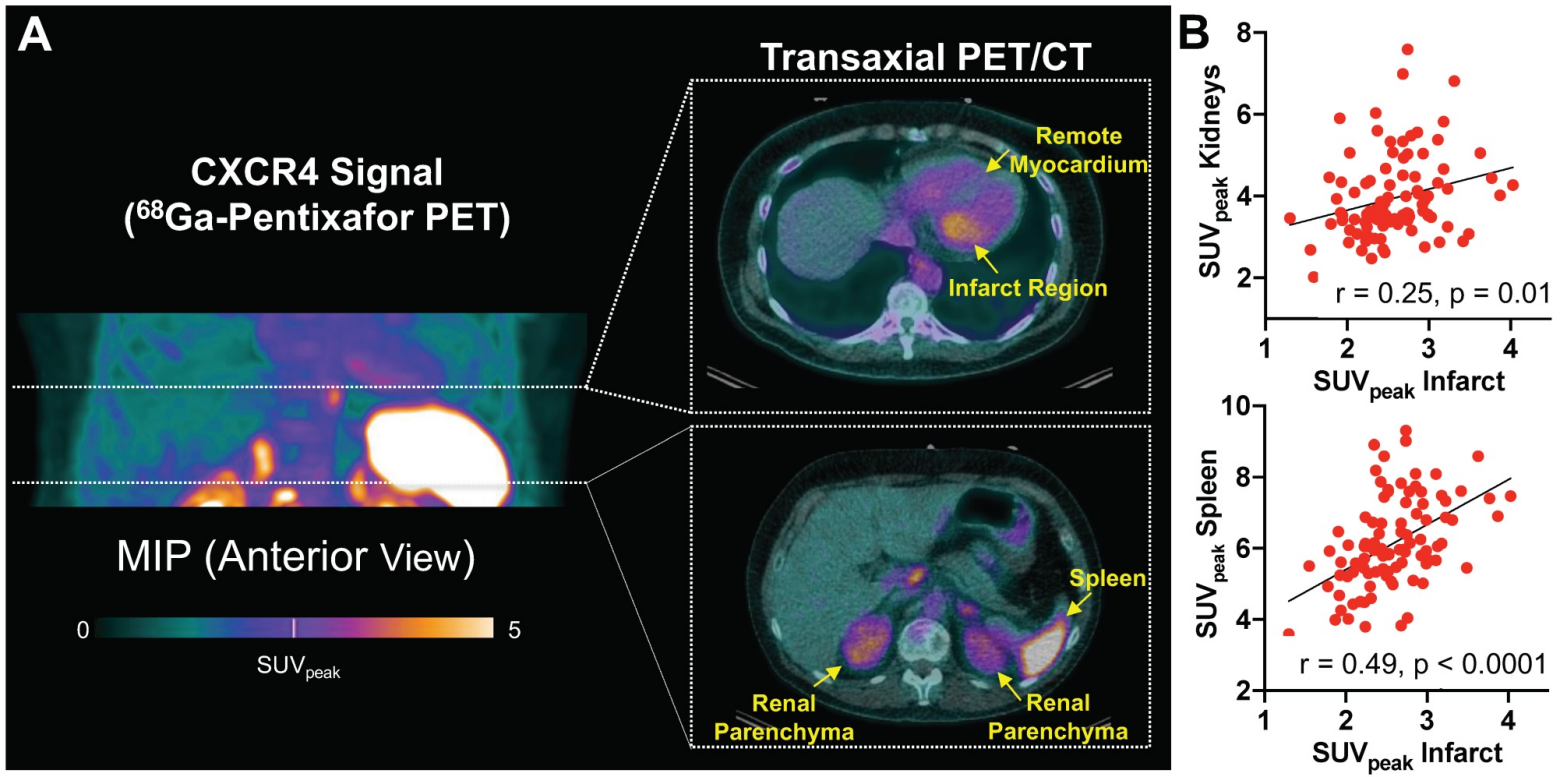
(A) Maximum intensity (MIP) projection of ^68^Ga-Pentixafor PET in a patient early after acute myocardial infarction, along with representative transaxial hybrid PET/CT slices. Spherical volumes of interest are placed in infarct and remote myocardium, spleen, and renal parenchyma. (B) CXCR4 signal derived from infarct territory correlated with uptake in renal parenchyma and spleen. SUV_peak_ = peak standardized uptake value (n = 96).

To investigate translational potential, we applied a similar analytical approach in a series of patients after myocardial infarction. CXCR4 PET signal from heart, renal parenchyma and hematopoietic organs were identified in patients after reperfused AMI (**Figure 4A**). ^68^Ga-Pentixafor signal from infarct was significantly higher when compared to remote myocardium (infarct/blood pool *vs.* remote/blood pool ratios, 1.26 ± 0.23 *vs.* 0.75 ± 0.17, p < 0.0001). CXCR4 signal intensity from renal parenchyma correlated with the signal from infarct (r = 0.25, p=0.01) (**Figure 4B**) and remote myocardium (r = 0.39, p < 0.0001), indicating an inflammatory link between heart and kidneys across species. As observed in mice, signal from the spleen also correlated significantly with infarct (r = 0.49, p < 0.0001) (**Figure 4B**) and renal signal (r = 0.36, p < 0.001), further supporting the notion of a systemic network in patients after AMI.

### Cardiac CXCR4 signal predicts adverse renal outcome in AMI patients

**Figure 5:**
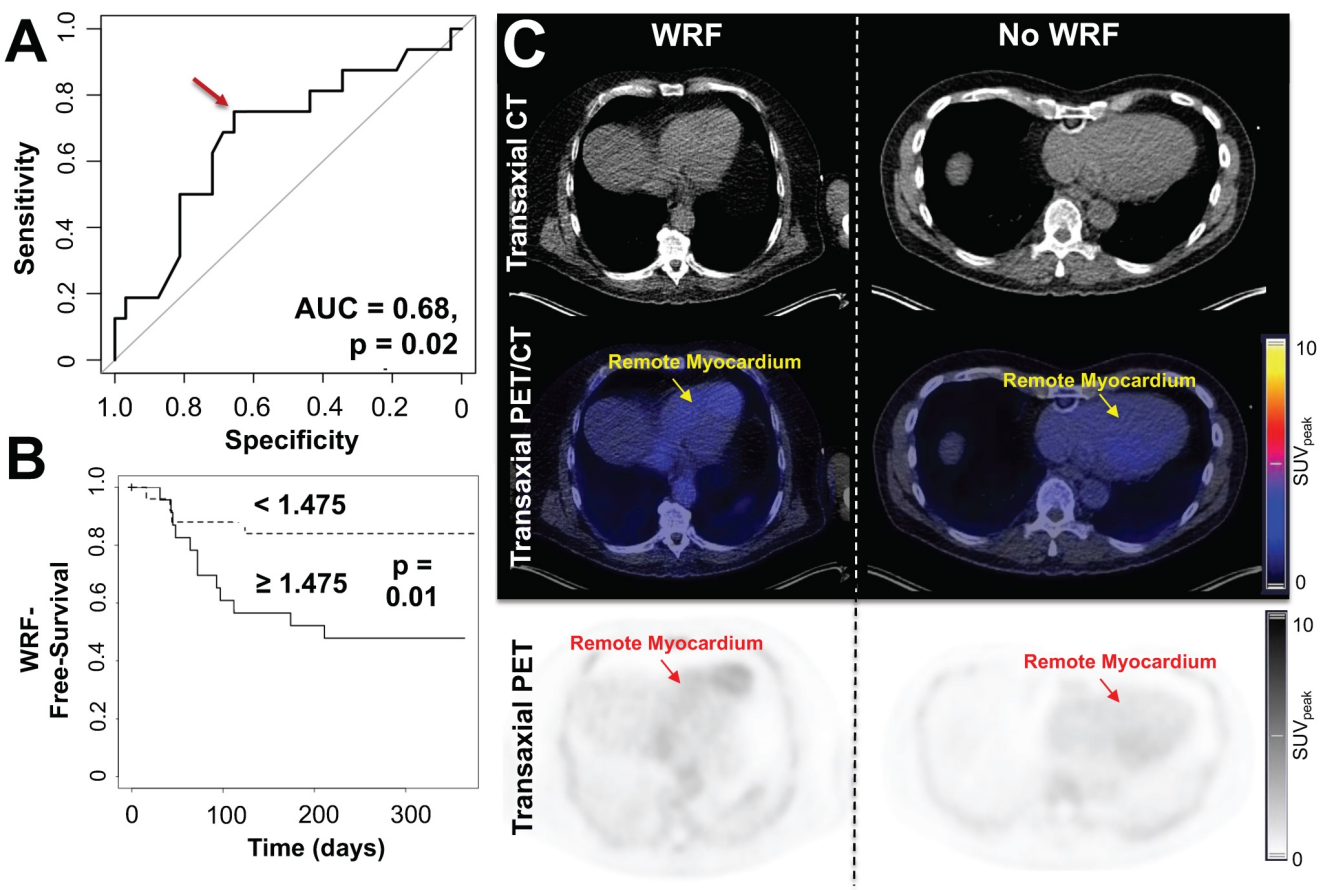
(A) Receiver-operating-characteristics (ROC) curve for the association between CXCR4 tracer signal in remote myocardium and worsening of renal function (WRF) occurrence. Optimal cutoff is defined by a red arrow. (B) Kaplan-Meier curves for WRF-free survival in patient groups with increased *vs.* decreased myocardial remote CXCR4 signal. WRF during follow-up occurred primarily in patients showing increased CXCR4 uptake in the remote myocardium, while in subjects with no further decline in renal function, no discernible radiotracer signal in the cardiac remote area was noted. (C) Transaxial computed tomography (CT), positron emission tomography (PET)/CT and PET images of patients after myocardial infarction with (left) and without worsening of renal function (WRF, right). Left patient showed increased CXCR4 signal in the remote myocardium and a loss of glomerular filtration rate (GFR) of 6.2 mL/min/1.73 m^2^ was noted during follow-up. Right patient had no decline in renal function (GFR loss, 0.6 mL/min/1.73 m^2^) and demonstrated no substantial elevated CXCR4 expression in the remote myocardium. SUV_peak_ = peak standardized uptake value. (n = 48).

At the time of PET, GFR did not correlate with CXCR4 signal in any organ (r ≤ 0.04, n.s.). During follow-up, GFR was available in 48/96 subjects (50%) (**Table 1**) and declined from 85 ± 19 to 80 ± 19 mL/min/1.73 m^2^. In 16/48 (33.3%), the predefined criteria for worsening renal function were met at a mean of 80.5 d post-MI. These patients exhibited significantly higher CXCR4 signal in remote myocardium compared to patients with no adverse renal outcome (SUV, 1.6 ± 0.38 vs no event, 1.37 ± 0.32, p = 0.03) (**Table 2**). On ROC analysis, GFR at baseline (AUC = 0.49, p = 0.47), PET-derived CXCR4 signal from the infarct (AUC = 0.55, p = 0.29), remote/blood pool ratios (AUC = 0.56, p = 0.24), infarct/blood pool ratios (AUC = 0.59, p = 0.15), and PET-based kidney signal (AUC = 0.59, p=0.16) were not significantly associated with presence of worsening renal function. CXCR4 signal from remote myocardium, however, reached significance on ROC analysis (AUC = 0.68, p = 0.02, **Figure 5A**), which identified an optimal cutoff of 1.475 for highest accuracy. Using this ROC-derived cutoff, univariate Kaplan-Meier analysis revealed significantly impaired outcome for the subgroup with increased *vs.* decreased signal (p = 0.01, **Figure 5B-C**). Finally, when multivariate Cox regression was performed to include established clinical predictors such as baseline GFR, LVEF and infarct size, the remote myocardial signal remained the only independent predictor of renal events (Hazard Ratio 5.27, p = 0.03), yielding an event probability of 84.1% (**Table 3**).

## Discussion

In summary, our work based on whole-body, molecular imaging suggests that immune-mediated mechanisms play a role in cardio-renal crosstalk early after myocardial injury, and impacts subsequent functional outcome in both organs. Our results confirm the feasibility of assessing cardiorenal inflammatory interaction in a dedicated murine AMI model. Cardiac and renal signal were directly correlated, suggesting an inflammatory link between heart and kidneys, and *ex-vivo* tissue work-up confirmed the time course and inflammatory origin of the radiotracer signal in both organs in a longitudinal setting. Emphasizing its predictive potential to identify adverse cardiac functional outcome, early *in-vivo* CXCR4 signal in the kidneys was also inversely correlated to independently assessed late LVEF in mice. Of note, a close interaction of heart and kidneys was also observed in CXCR4 PET studies of patients after AMI, confirming the translational potential of the murine observations.

CXCR4 and its ligand, stromal-cell derived factor C-X-C motif chemokine 12, tightly orchestrate the repair of injured tissue in general, and the myocardium after MI specifically 21. A growing body of evidence has reported on the use of the radiolabeled CXCR4 ligand ^68^Ga-Pentixafor that allows for high-contrast visualization of the presence of the receptor in the infarct territory *in-vivo* 13. Seeking for imaging-guided therapeutic interventions, CXCR4 blockade improved late LVEF when applied during PET-guided peak myocardial CXCR4 upregulation, but not off-peak 22. Further supporting the concept of image-based guidance for timing initiation of CXCR4 blockade, Jujo *et al.* demonstrated that a single-dose injection of the small molecule CXCR4 antagonist AMD3100 administered after the onset of MI in mice improved cardiac function, whereas continuous therapy with AMD3100 was associated with worse outcome 23. Paving the way for clinical trials, the clinical stage CXCR4 antagonist POL6326 also led to an improvement of mechanical function after acute MI in pigs 24. Nonetheless, the dichotomous nature of CXCR4 during the pathogenesis and evolution of MI has to be considered. Liehn *et al.* demonstrated in a permanent occlusion model that infarct size was smaller in CXCR4^+/-^ mice relative to wild-type mice along with decreased neutrophil content and adaptation to hypoxic stress, while angiogenesis was impaired. Therefore, such a double-edged role of CXR4 should be taken into account when introducing CXCR4-targeted molecular image-guided therapeutic interventions 25.

This work confirmed the importance of CXCR4 as a target for reparative therapy and imaging in the heart. Yet, information on its link with the kidneys remains scarce. A recent translational study investigating *ex-vivo* CXCR4 expression in a rat model of primary renal injury reported an increase on endothelial cells in both the glomerular and peritubular compartments 26. This is consistent with the *in-vivo* and *ex-vivo* radiotracer accumulation throughout the entire kidney parenchyma in the present study. Moreover, similar to the myocardium, where continuous infusion of CXCR4 antagonists in mice after AMI exacerbated cardiac dysfunction 23, chronic CXCR4 blockade in both rats and humans afflicted with aberration of the renal microvessels also led to augmented renal decline and progressive renal fibrosis 26. The present study confirms that acute cardiac injury leads to an elevated CXCR4-related inflammation signal not only in the heart, but also in the kidneys, with importance for the time course of function in both organs. A next step may be to explore strategies for CXCR4-targeted imaging to guide targeted interventions for restoration of bilateral heart-kidney function to the right individual and the right time. These considerations are further fueled by recent recommendations of the American Heart Association Council which emphasized the need for novel imaging technologies to improve future clinical practice in cardio-renal medicine, preferably by implementing strategies that guide towards novel targeted therapies 2.

For patients with AMI and subsequent ventricular remodeling leading to heart failure, the primary myocardial injury has been shown to trigger worsening of renal function in some subjects, which in turn accelerates the risk of hospitalization and cardiac death 2. Thus, representing the most important comorbidity in a post-AMI setting, strategies identifying patients at increased risk for renal impairment are urgently needed. In heart failure, a recent meta-analysis identified baseline renal deterioration as the main risk factor for development of renal decline 27. The Acute Coronary Treatment and Intervention Outcomes Network (ACTION) Registry investigated patients early after AMI and reported on impaired serum creatinine as one of the attributable risks associated with onset of worsening renal function 28. In our clinical cohort of optimally treated ST-segment elevated AMI patients, cardiac CXCR4 expression outperformed established clinical predictors, including baseline GFR, LVEF or infarct size in identifying patients which are prone to later renal impairment. This observation suggests that a direct visualization of the inflammatory cell content in the myocardium may have better predictive value compared to commonly used clinical parameters to predict worsening of renal function. As such, one may speculate that the concept of targeted inflammatory molecular imaging of the myocardium can be expanded towards interrogation of reciprocal networking between heart and kidney, thereby suggesting an additional role for imaging-guided, renoprotective strategies early after MI 29.

In addition, Ruparelia and coworkers reported on *ex-vivo* derived cytokine upregulation in the infarct, the kidneys and in remote myocardium, thereby indicating a systemic inflammatory response post-MI 30. Further expanding on these findings by using molecular imaging, Lee *et al.* recently investigated MI in mice using ^18^F-FDG PET and reported on an increased signal in the non-infarcted remote myocardium relative to control hearts, along with leukocyte trafficking in cardiac areas remote from the site of ischemic damage 31. Of note, assessed by fluorescence-activated cell sorting, CD11b^+^ cells were lower in the remote myocardium right after AMI, but reached a peak later in the timecourse which was even superior relative to the infarcted area. While conclusions from mice to humans have to been drawn with caution, not all patients enrolled in the present study have been imaged right after the acute event. Therefore, given the findings of Lee and coworkers 31, this may explain the superior role of remote myocardial uptake for adverse renal outcome relative to radiotracer accumulation in the infarct in the present study. Thus, in future investigations, imaging directly after guideline-compatible treatment post-MI may demonstrate a superior predictive performance of the infarct territory for adverse renal outcome when compared to remote sites of ischemic injury. Moreover, in mice, the early infarct signal at MI+1d or MI+3d predicted worse cardiac functional outcome at 6 weeks 12. However, in the present study, renal CXCR4 signal at MI+7d emerged as a stronger predictor for later adverse cardiac functional outcome relative to the infarct signal at the same day post-MI, potentially reflecting a different timeframe to the kidney damage relating to cardiac dysfunction. Thus, those differences in the predictive values of the CXCR4 signal derived from both organs may be interpreted as a response to worsening cardiac function and remodeling that leads to the subsequent inflammatory cell content in the kidney.

### Study limitations

First, although activated macrophages seem to be critically involved, the exact inflammatory cell population contributing to the *in-vivo* renal and cardiac CXCR4 signal cannot be identified. Thus, further studies should provide more precise information on the different leukocyte subtype profiles contributing to the distinct phases of the inflammatory pathway along the cardiorenal axis. It should be noted that ^68^Ga-Pentixafor has a lower affinity for murine CXCR4 than for the human variant, which may influence the amplitude of absolute tracer uptake. But specific binding and response to therapy are well established 12, 13. While murine-specific imaging agents have been reported 32, significant liver activity is not conducive to cardiac or renal imaging of CXCR4 in mice. In addition, ^68^Ga-labeled radiotracers have a high kinetic energy and this may impact image quality and degrade diagnostic yield and therefore, ^18^F-labeled CXCR4 radiotracer may overcome this issue 33. Moreover, a permanent coronary occlusion model was chosen in the present feasibility study, as this provides more consistent infarcts relative to reperfusion injury 34 and leads to heart failure 35. Future studies, however, may also investigate the impact of occlusion-reperfusion along the cardiorenal axis. The non-specific contribution to the *in vivo* CXCR4 signal in the kidneys (spill-in/-over) may be more clearly specified in future studies, e.g. by performing a (hemo)dynamic dual-radiotracer approach using inflammation targeting and functional renal imaging agents, preferably by enrolling a control group without MI 36. Autoradiography of rinsed renal parenchyma and histologic identification of elevated CD68-positive leukocytes, however, supports the notion that the renal *in vivo* imaging signal cannot be attributed to impaired renal excretion only. Renal CXCR4 *in-vivo* signal could be further corroborated by conducting a more specific *ex-vivo* analysis, e.g. by investigating leukocyte-recruiting vascular cell adhesion molecule 1 and CD68 in the kidneys in a longitudinal setting 30, preferably in a larger set of animals. Such an approach would allow to draw more meaningful conclusions whether elevated CXCR4 signal indeed corresponds to increased macrophage density in infarct territory and kidneys. In patients, GFR at baseline was also not associated with renal CXCR4 signal, which is in line with a previous study investigating subjects with renal allograft infections 37. Future studies investigating the cardiorenal axis, however, could also apply dedicated diuretic protocols to facilitate assessment of the kidneys imaged with ^68^Ga-Pentixafor 37. Moreover, although mice and patients with primary cardiac injury were investigated, we herein report on the role of renal CXCR4 uptake for late cardiac functional outcome in mice and the impact of cardiac CXCR4 signal for worsening renal function in humans. As a consequence, whole-body inflammatory PET should be also applied to explore renal impairment in animals after AMI and functional cardiac decline in AMI patients. Moreover, similar to previous reports on worsening renal function in the post-MI setting, the present proof-of-concept study investigated a decrease in estimated GFR. Nonetheless, fluctuations of GFR are not always necessarily indicative of prognosis 19, and thus, the reported predictive capability of CXCR4 PET for occurrence of renal deterioration should be interpreted with caution. Future studies may therefore incorporate a more rigorous definition of late renal impairment, e.g. by including blood urea nitrogen or cystatin C, preferably with a repeated number of measurements during follow-up 27, which may then reveal a superior prognostic performance of the infarcted or remote myocardium for adverse renal outcome.

## Conclusions

In conclusion, CXCR4-targeted whole-body molecular imaging revealed tight inflammatory interaction between the myocardium and the kidneys as secondary affected organs after primary cardiac injury. AMI leads to an early inflammatory immune response in the infarct territory followed by a rapid decline, which was paralleled by temporal inflammation in the kidneys post-MI. In a translational approach investigating optimally treated AMI patients, cardiac CXCR4-directed PET imaging also outperformed established clinical parameters in identifying patients at increased risk for worsening renal function, which may bear potential for image-based guidance of renoprotective strategies early after AMI. Taken together, a systems-based molecular imaging-based network analysis may therefore increase the individual benefit by guiding therapy not only based on the disease state in the target organ, but also in networking other tissues.

## Supplementary Material

Supplementary figure.

## Figures and Tables

**Table 1:** Clinical characteristics of patients with available glomerular filtration rate during follow-up (n = 48)

Variable	Data
Age (y)	61 ± 11
**Sex**	
Female	7/48 (14.6)
Male	41/48 (85.4)
**Risk factors**	
History of Smoking	28/48 (58.3)
History of Diabetes	13/48 (27.1)
History of Hypertension	24/48 (50)
History of Hyperlipidemia	23/48 (47.9)
History of Obesity	10/48 (20.8)
**Medication at PET**	
Oral Antidiabetics or Insulin	11/48 (22.9)
Angiotensin-Converting Enzyme Inhibitor	41/48 (85.4)
Angiotensin Receptor Blocker	7/48 (14.6)

Data are mean ± SD. Data in parentheses are percentages.

**Table 2:** ^68^Ga-Pentixafor positron emission tomography-derived and clinical parameters for patients with or without worsening renal function (WRF)

WRF	Yes (n=16)	No (n=32)	p-value
***^68^Ga-Pentixafor Signal***			
Remote Myocardium*	1.6 ± 0.38	1.37 ± 0.32	0.03
Renal Parenchyma	4.36 ± 1.25	3.96 ± 0.81	0.18
Global Myocardium	2.07 ± 0.38	1.92 ± 0.34	0.18
Infarct	2.50 ± 0.46	2.48 ± 0.49	0.67
Spleen	5.76 ± 1.17	5.93 ± 1.34	0.68
Infarct/Blood Pool Ratio	1.24 ± 0.22	1.32 ± 0.20	0.84
Remote Myocardium/Blood Pool Ratio	0.77 ± 0.14	0.73 ± 0.17	0.48
***Clinical Parameters***			
LVEF (%)	48.20 ± 8.73	50.53 ± 13.20	0.54
Infarct Size	11.19 ± 9.61	9.97 ± 7.63	0.63
GFR at baseline (mL/min/1.73 m^2^)	85.58 ± 20.09	84.43 ± 18.47	0.84

*reached significance.

**Table 3:** Results of multivariate cox regression analysis assessing relationship between clinical parameters, ^68^Ga-Pentixafor positron emission tomography-derived signal in the remote myocardium and outcome (worsening renal function)

Variable	Hazard Ratio	95% Confidence interval	p-value
Remote Myocardium*	5.27	1.19-23.30	0.03
GFR at baseline (mL/min/1.73 m^2^)	1.02	0.98-1.05	0.27
Infarct Size	1.02	0.98-1.08	0.47
LVEF	0.99	0.94-1.03	0.6

GFR, glomerular filtration rate; LVEF, left ventricular ejection fraction. * reached significance.

## Abbreviations

AMI: acute myocardial infarction
CXCR4: CXC motif chemokine receptor 4
GFR: glomerular filtration rate
PET: positron emission tomography
SPECT: single-photon emission computed tomography
SUV: standardized uptake values
