# Supplementary material for: Molecular imaging of inflammation crosstalk along the cardio-renal axis following acute myocardial infarction
Source: Theranostics. 2021 Jul 6;11(16):7984–94. doi: 10.7150/thno.61423 (PMC8315063; doi:10.7150/thno.61423)
Supplement: Supplementary file 1 — Supplementary figure. [file thnov11p7984s1.pdf]

Figure S1. (A) In animals with severe left ventricular dysfunction (LVEF < 30%), cardiac (A, n=8) and renal (B, n = 7) signal obtained one day after myocardial infarction (MI+1d) did not reach significance with late cardiac function at 6 weeks. In a similar manner, cardiac (C, n = 9) or renal (D, n = 8) signal at MI+3d was also not significantly associated with late cardiac function 6 weeks after MI. Mice with modest cardiac dysfunction (LVEF > 30%) displayed no correlation between subacute kidney signal at MI+7d and late ejection fraction 6 weeks post-MI (E, n = 10).

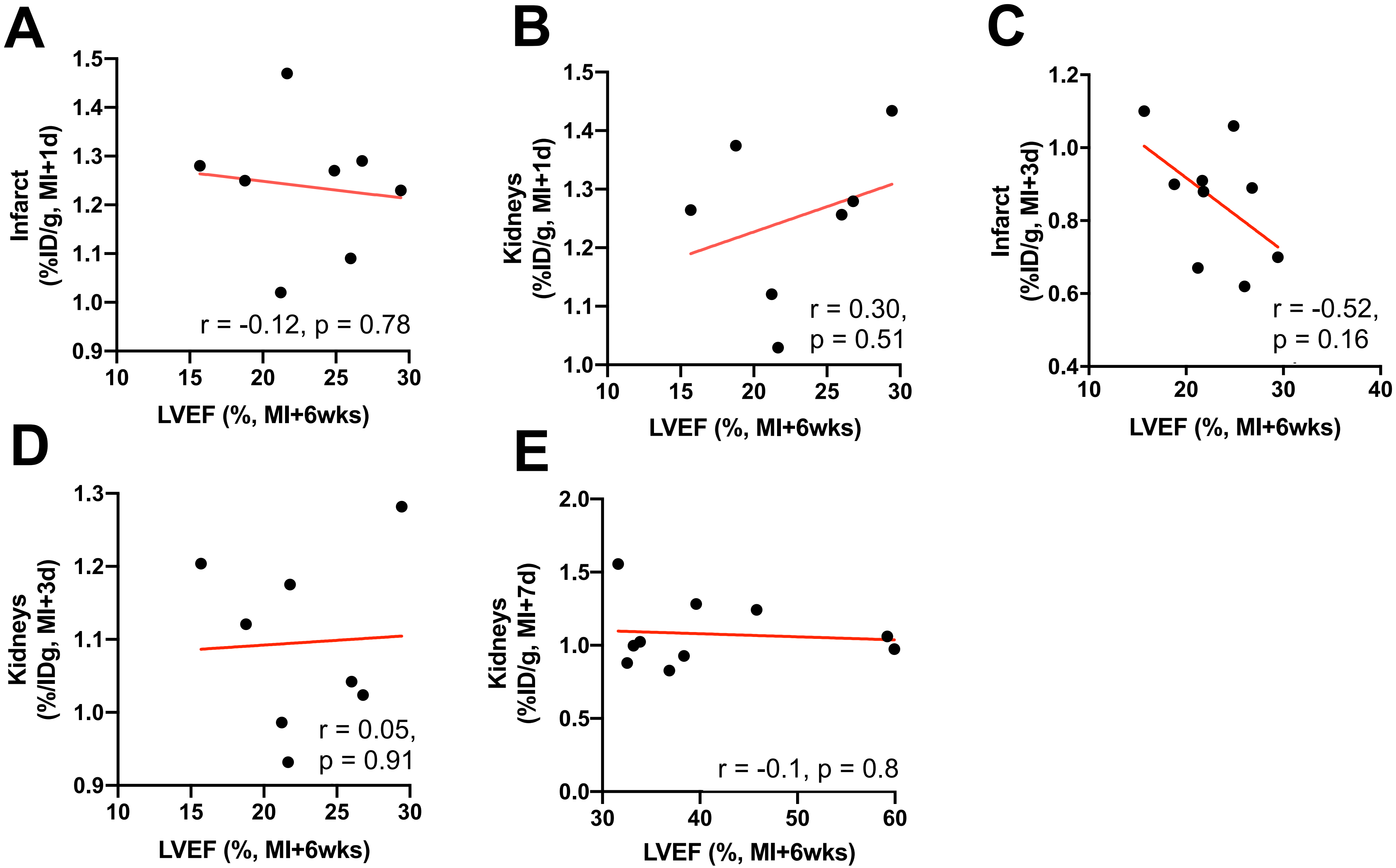

A
Infarct (%ID/g, MI+1d)
r = -0.12, p = 0.78
LVEF (%, MI+6wks)
B
Kidneys (%ID/g, MI+1d)
r = 0.30, p = 0.51
LVEF (%, MI+6wks)
C
Infarct (%ID/g, MI+3d)
r = -0.52, p = 0.16
LVEF (%, MI+6wks)
D
Kidneys (%/IDg, MI+3d)
r = 0.05, p = 0.91
LVEF (%, MI+6wks)
E
Kidneys (%ID/g, MI+7d)
r = -0.1, p = 0.8
LVEF (%, MI+6wks)
